# Delayed Formation of Traumatic Ulnar Artery Pseudoaneurysm Presenting With Ulnar Nerve Palsy: A Case Report

**DOI:** 10.7759/cureus.28744

**Published:** 2022-09-03

**Authors:** Russell Seth Martins, Ali Abdullah Gill, Baila Maqbool

**Affiliations:** 1 CITRIC Center for Clinical Best Practices, Aga Khan University, Karachi, PAK; 2 Department of Surgery, University of New Mexico School of Medicine, Albuquerque, USA

**Keywords:** compartment syndrome, hematoma, pseudoaneurysm, trauma, ulnar artery

## Abstract

Ulnar artery pseudoaneurysm (UAP) is a rare occurrence after penetrating injury to the distal upper extremity and may lead to complications such as rupture, sensorimotor dysfunction, and compartment syndrome. We present the case of a 57-year-old man who developed delayed UAP after suffering a penetrating injury to the right forearm. UAP was diagnosed a week after the injury using CT angiography (CTA), which was indicated due to the onset of ulnar nerve palsy (mildly reduced hand-grip strength and fourth- and fifth-digit hypoesthesia and numbness) and growing swelling and tenderness of the right forearm. Due to concerns about UAP and hematoma formation, with resultant compression of the ulnar nerve and suspected hematoma infection, surgical intervention was performed wherein a moderate-size hematoma was evacuated and the ulnar artery was ligated. The decision to ligate rather than reconstruct was based on the suspected infected nature of the hematoma and adequate perfusion of the palmar arch by the radial artery. This case highlights the need for maintaining a strong index of suspicion for UAP after penetrating trauma to the distal upper extremity, due to the possibility of debilitating nerve deficits and compartment syndrome complicating late diagnosis. We also present an algorithm for the choice of management modality for UAP, which is a valuable addition to the existing literature.

## Introduction

Ulnar artery pseudoaneurysm (UAP) is a rare entity. Although its true natural incidence is unknown, it has been reported to develop in 0.1% of patients after endovascular interventions requiring arterial puncture [1]. While a true aneurysm entails an outpouching of all three layers of the vessel wall, a pseudoaneurysm consists of a pocket of blood contained within either a single adventitial layer or an extraluminal wall developed by coagulation cascade products (fibrin-platelet mesh) [2,3]. Pseudoaneurysms mostly arise in the femoral, aortic, or visceral arteries [2], with upper extremity pseudoaneurysm, particularly UAP, being extremely rare (1%). They mostly arise due to blunt or penetrating trauma, iatrogenic causes, and atherosclerotic or degenerative lesions [4]. Sequela may include local compression of surrounding structures, embolization, or rupture [2].

Most cases of UAP are due to repeated, chronic microtrauma, with very few of the reported cases being acutely post-traumatic. In rare cases, pseudoaneurysms may present with years of delay after the complete healing of an acute traumatic injury [5]. Surgery remains the mainstay of treatment for UAP, although newer therapeutics are being explored. Complications of UAP include thrombosis, distal embolic disease, and ulnar nerve compression [6,7]. Rupture of a UAP is exceedingly uncommon, but its occurrence may lead to acute neurologic compromise [8]. In this report, we discuss a case of missed acute post-traumatic UAP with rupture and hematoma formation, in accordance with the 2018 SCARE guidelines [9]. We also present an algorithm for the choice of management modality for UAP, which is a valuable addition to the existing literature.

## Case presentation

Initial presentation

A 57-year-old male was transported by emergency medical services (EMS) to the ER of a tertiary care hospital in Albuquerque, New Mexico. The patient had suffered a self-inflicted stab wound to the right forearm, which had occurred when he had accidentally lacerated his forearm deeply on a fish fillet knife. He was hemodynamically stable and grossly intact neurologically.

On initial evaluation by the trauma surgery service, the patient had intact upper and lower extremity pulses bilaterally, including bilateral radial and ulnar pulses. The laceration on the medial right forearm measured 3 cm in length, with brisk bleeding. Hemostatic sutures (3.0 Vicryl) were placed in the laceration to stop the bleeding. The hand continued to be well-perfused and warm. CT angiography (CTA) was performed, which revealed no major vascular injury. The laceration was copiously irrigated and did not bleed any further and was closed with three interrupted nylon sutures.

The patient had a history of anxiety and was taking appropriate prescription medications. There was no significant medical or surgical history, allergies, history of substance use, or family history of bleeding disorders. Laboratory results for serum lactate, prothrombin time, international normalized ratio, fibrinogen, platelet count, and hemoglobin (16.4 g/dL) were within normal ranges. The patient remained hemodynamically stable over the next 30 hours, with no signs of compartment syndrome.

Second visit

The patient presented to the ER again one week later, with complaints of increasing swelling, tenderness, and tension of the right forearm with erythema that had developed over the past week. He developed hypoesthesia of the fourth and fifth digits on the right hand as well, with decreased range of motion (ROM) of the wrist, hand, and fingers, due to the pain and swelling. There was no history of fevers or chills or other pertinent symptoms, and a review of systems was unremarkable.

The patient was hemodynamically stable. The right forearm was edematous, erythematous, tense, and tender to superficial palpation, with a well-approximated previously sutured laceration. Vascular examination revealed a bounding right ulnar pulse, with a normal palpable right radial pulse and well-perfused hand. Apart from a hemoglobin level of 11.4 g/dL (which was decreased in comparison to a week ago), laboratory tests including a complete blood count and serum electrolytes were unremarkable.

**Figure 1:**
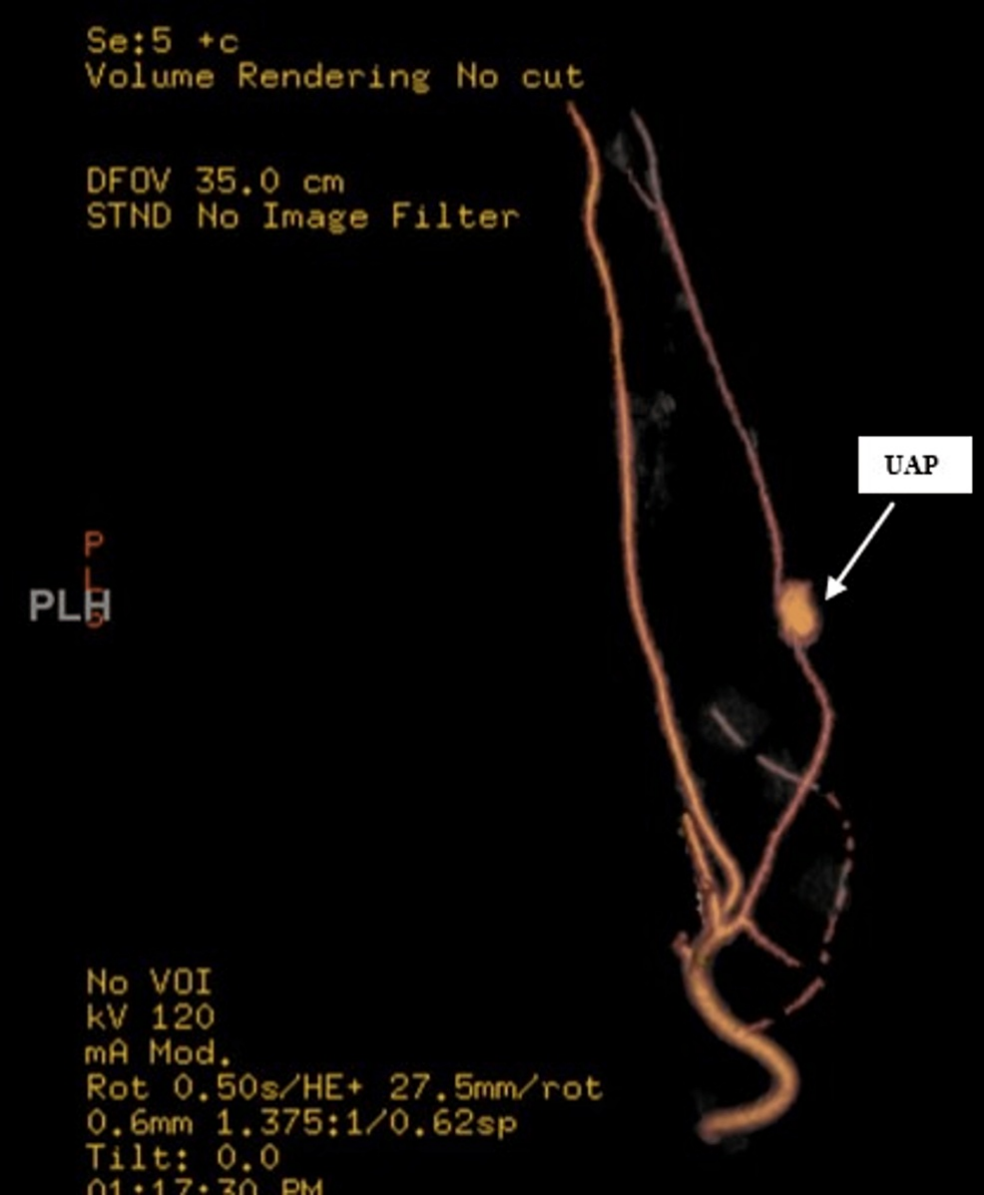
CTA demonstrating focal collection concerning for pseudoaneurysm CTA: computed tomography angiography

CTA performed at a referring center showed a focal collection of extravascular contrast (1.6 x 0.9 mm) along the ulnar artery in the right mid-forearm, suggestive of right UAP with surrounding hematoma (Figure 1).

Based on these physical and radiologic findings, there was a concern about delayed UAP formation secondary to recent trauma, superimposed with cellulitis, and a possible infected hematoma. Intravenous antibiotics (vancomycin and piperacillin/tazobactam) were started. Eventually, an operative exploration was planned. The decision to opt for a surgical approach, as opposed to conservative thrombin injection, endovascular stent-graft placement, or external compression, was driven by the threat of critical neurovascular compression and possible infection.

Operative details

Prior to the operation, the patient's vascular flow to the hand was assessed by occluding the ulnar artery and appreciating good Doppler signals at the palmar arches and digits. The palmar arch was well-perfused by the radial artery. The operation was initiated by opening the prior wound closure. A moderate-volume hematoma was found tracking from the mid-forearm wound to the proximal forearm, which was evacuated. A swab of the wound was taken for culture and sensitivity. The ulnar artery demonstrated an active arterial bleed from damaged proximal and distal ends, and a 3-4-cm area of the artery appeared shredded in appearance, representing a ruptured pseudoaneurysm. Dissection was continued to enable the visualization of the proximal and distal ends of the ulnar artery, where it was ligated. Other small branches, as well as bleeding muscular vessels, were ligated. The decision to ligate rather than reconstruct was based on the suspected infected nature of the hematoma and adequate perfusion of the palmar arch by the radial artery. After the ligation of the ulnar artery, the patient still had a palpable radial pulse, and positive Doppler signals at the radial position, both palmar arches, and at all five digits. Local wound hemostasis was achieved, after which the tourniquet was removed, and hemostasis was confirmed.

**Figure 2:**
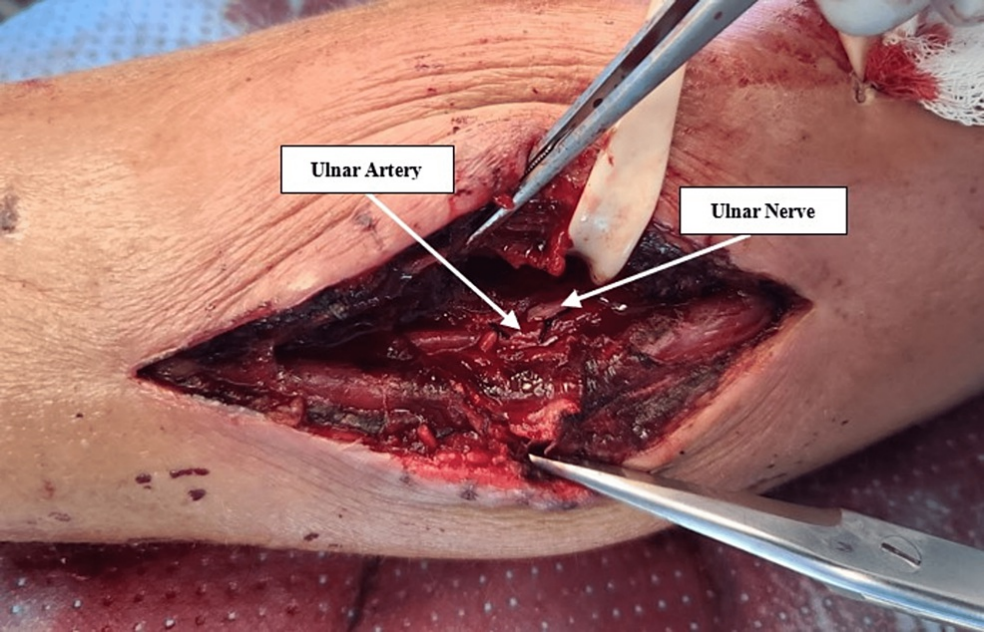
Operative findings showing ligated ulnar artery and its proximity to the ulnar nerve after hematoma evacuation

The ulnar nerve was identified and protected, and no obvious injury was observed. The area of the hematoma was copiously irrigated (Figure 2). A drain was left in place and the wound was closed primarily again. The patient remained hemodynamically stable throughout the operation. Postoperatively, oxygen saturations in the hand on the affected limb were found to be normal. Two days later, just prior to discharge, his wound culture grew methicillin-resistant *Staphylococcus aureus* (MRSA). Following this, the patient was started on a week’s course of oral doxycycline and was discharged in stable condition on the second postoperative day, with the hand warm and well-perfused.

Postoperative follow-up

At the follow-up appointment six days later, the patient’s forearm pain had resolved the hand grip strength was 5/5, and the numbness to the fourth and fifth digits on the right hand was improving. On examination, the right radial pulse was strong and palpable, all fingers were warm and well-perfused, and there was a minor limitation to the ROM of the right hand. The right forearm laceration demonstrated healing with no surrounding erythema or drainage, indicating abatement of the suspected infection.

## Discussion

We presented the case of a 57-year-old male who developed UAP in a delayed manner after penetrating trauma, with sensorimotor ulnar nerve palsy, which subsequently led to a rupture and hematoma formation. The patient was operatively managed, which entailed the evacuation of the hematoma and ligation repair of the ulnar artery. Ulnar nerve deficits included a mildly reduced hand-grip strength and fourth- and fifth-digit hypoesthesia and numbness.

Acute post-traumatic UAP should be suspected in patients presenting with a pulsatile, palpable, and painful mass that is well-limited and compressible [5]. However, our patient presented with an area of localized swelling on the forearm, as opposed to a discernible mass. Symptoms that may accompany the presentation of UAP include those of distal hypoperfusion or compression of nearby nerves [5]. Compression of the ulnar nerve is the most commonly observed one, with resulting sensory and motor neuropathy, as in our patient. Neuropathies may be particularly concerning in the event of acute rupture of UAP, and rapid intervention is indicated in such cases [5]. Both sensory and motor deficits of the ulnar nerve tend to resolve after early repair of the UAP [10]. If missed on initial presentation, UAP may lead to features of compartment syndrome, with debilitating and possibly permanent neurovascular complications. Given the increased swelling, tension, and tenderness developing a week after the initial injury in our patient, it is likely that our surgical intervention was performed not a moment too soon.

Diagnostic imaging modalities for UAP include selective upper extremity arteriography, ultrasonography, MRI, and CTA. Ultrasonographic features include a hypoechoic saccular cystic formation demonstrating turbulent internal blood flow and arising from the adjacent ulnar artery [11]. The classic finding on Doppler ultrasound is the “yin-yang” sign (a swirl of colors caused by the bidirectional flow within the UAP) [3]. The most specific sign of a pseudoaneurysm is the “to and fro” waveform on duplex ultrasound, seen due to the communicating channel between the artery and the pseudoaneurysmal sac [12]. However, ultrasonography may still be limited due to being operator-dependent. MRI, although highly sensitive and specific, is precluded from being a viable routine imaging option because of its time-intensive and expensive nature. Interestingly, Kehara et al. have described a case of nontraumatic UAP that resembled a soft-tissue tumor on CT and MRI due to atypical imaging characteristics of the UAP [13].

In our case, CTA provided evidence of a UAP with surrounding hematoma. CTA is an extremely valuable tool in the evaluation of UAP, as it is quickly obtainable, has a highly specific and sensitive diagnostic ability, can detect active extravasation, and assists in surgical planning [3,12]. It is steadily growing in favor as compared to selective upper extremity arteriography, the long-accepted gold standard investigation for diagnosis [14]. Nevertheless, although catheter arteriography is becoming less and less common as the initial diagnostic imaging of choice, it is a useful tool when CTA findings are inconclusive or when endovascular intervention is due to be performed [15].

**Figure 3:**
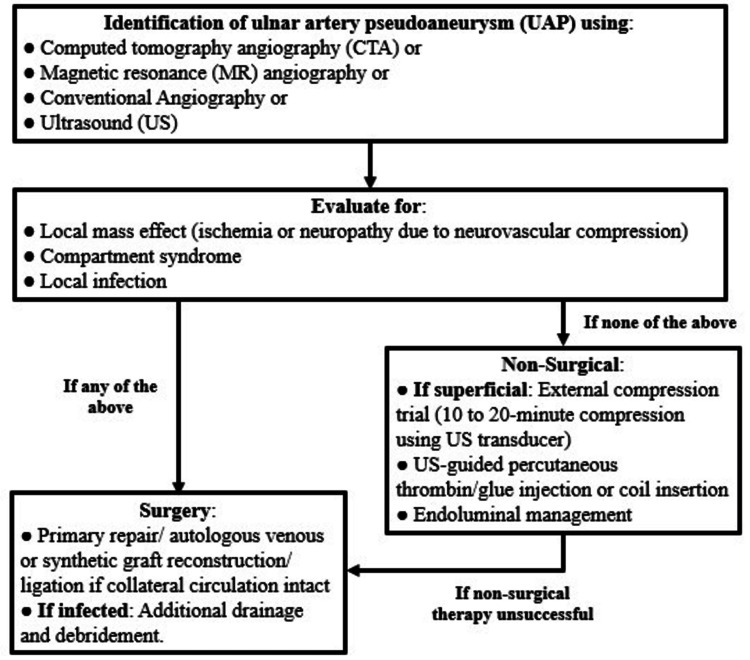
Algorithm for deciding on the management approach to ulnar artery pseudoaneurysm

To the best of our knowledge, there is currently no algorithm delineating the choice of management modality for UAP. Surgical management was opted for in our case, a decision guided by the ulnar nerve motor and sensory deficits, growing swelling and tenderness, and suspicion of infection. Surgery for UAP is indicated in cases where it is causing neurovascular compression, or if non-surgical treatment has failed. Surgical intervention is particularly urgent in cases where there is evidence of infection, active extravasation, significant erosion of the overlying skin, or signs of compartment syndrome of the affected extremity [16]. Surgical techniques include excision of the UAP with arterial ligation, followed by revascularization using direct suture anastomosis or a bypass procedure, or removal of the organ within which the pseudoaneurysm is contained [17]. Although surgery is the mainstay of treatment for UAP, ultrasound-guided thrombin injection is a novel therapeutic modality that has also been shown to be effective [18], although the use of thrombin injection for small-vessel aneurysm carries the risk of systemic intravascular thrombosis and distal ischemia [17]. Endovascular stent-graft placement, although infrequently performed, has also proved to be effective in the management of UAP [19]. Lastly, Vancabeke et al. have reported the use of manual compression with an elastic bandage to manage UAP conservatively [20], and ultrasound-guided compression (UGC) has been used with reasonable success for UAP [19]. However, surgery is indicated in cases of treatment failure with UGC [21]. Figure 3 shows our suggested algorithm for choosing the right approach to managing a UAP.

## Conclusions

Even in the absence of the classic palpable, pulsatile mass, UAP must remain within a clinician’s index of suspicion in the setting of non-resolving sensorimotor dysfunction after penetrating injury to the distal upper extremity. CTA is appropriate as an initial diagnostic modality, and urgent surgical intervention is warranted in the setting of suspected infection, ulnar nerve palsy, and possible compartment syndrome.
